# An assessment of the level of concern among hospital-based health-care workers regarding MERS outbreaks in Saudi Arabia

**DOI:** 10.1186/s12879-016-2096-8

**Published:** 2017-01-03

**Authors:** Mostafa A. Abolfotouh, Ali A. AlQarni, Suliman M. Al-Ghamdi, Mahmoud Salam, Mohammed H. Al-Assiri, Hanan H. Balkhy

**Affiliations:** 1 King Abdullah International Medical Research Center (KAIMRC), King Saud bin Abdulaziz University for Health Sciences (KSAU-HS), PO 22490, Riyadh, 11426 Kingdom of Saudi Arabia; 2 King Saud bin-Abdulaziz University for Health Sciences, Riyadh, Saudi Arabia; 3 King Abdulaziz Medical City, Ministry of National Guard-Health Affairs, Riyadh, Saudi Arabia

**Keywords:** Concern, Attitude, Health-care worker, MERS-CoV, Saudi Arabia

## Abstract

**Background:**

Middle East Respiratory Syndrome (MERS) is caused by MERS coronavirus (MERS-CoV). More than 80% of reported cases have occurred in Saudi Arabia, with a mortality exceeding 50%. Health-care workers (HCWs) are at risk of acquiring and transmitting this virus, so the concerns of HCWs in Saudi Arabia regarding MERS were evaluated.

**Methods:**

An anonymous, self-administered, previously validated questionnaire was given to 1031 HCWs at three tertiary hospitals in Saudi Arabia from October to December, 2014. Concerns regarding the disease, its severity and governmental efforts to contain it, as well as disease outcomes were assessed using 31 concern statements in five distinct domains. A total concern score was calculated for each HCW. Multiple regression analyses were used to identify predictors of high concern scores.

**Results:**

The average age of participants was 37.1 ± 9.0 years, 65.8% were married and 59.1% were nurses. The majority of respondents (70.4%) felt at risk of contracting a MERS-CoV infection at work, 69.1% felt threatened if a colleague contracted MERS-CoV, 60.9% felt obliged to care for patients infected with MERS-CoV and 87.8% did not feel safe at work using standard precautions. In addition, 87.7% believed that the government should isolate patients with MERS in specialized hospitals, 73.7% agreed with travel restriction to and from areas affected by MERS and 65.3% agreed with avoiding inviting expatriates from such areas. After adjustment for covariates, high concern scores were significantly associated with being a Saudi national (*p* < 0.001), a non-physician (*p* < 0.001) and working in the central region (*p* < 0.001).

**Conclusions:**

The majority of respondents reported concern regarding MERS-CoV infection from exposure at work. The overall level of concern may be influenced by previous experience of MERS outbreaks and related cultural issues. The concerns of HCWs may affect their overall effectiveness in an outbreak and should be addressed by incorporating management strategies in outbreak planning.

## Background

Middle East Respiratory Syndrome coronavirus (MERS-CoV) is a recently identified species of the betacorona virus genus [1]. The first known case of MERS occurred in Jeddah in western Saudi Arabia in June 2012, and the causative virus was identified by the Egyptian virologist Ali Mohamed Zaki [2]. Epidemiologists and virologists are now attempting to understand the characteristics of the virus and the clinical features of infected patients. As of February 17, 2016, MERS-CoV had reportedly infected 1638 people [with 80% of those cases in Saudi Arabia] and caused 587 deaths globally [3]. The World Health Organization (WHO) has called for collaboration and established a network of academic and public-health researchers within affected member states to characterize the geographic spread and timeline of the cases [4]. The ministries of health of nine countries in Europe and the Middle East, including Saudi Arabia, have launched aggressive surveillance strategies and infection-control campaigns to counteract the MERS epidemic [4].

Health-care workers (HCWs) are at the frontiers in battles against the emergence, spread, control and resolution of infectious outbreaks around the world. As the probable source of MERS-CoV, the Kingdom of Saudi Arabia has a large number of Saudi nationals and expatriate health workers who are now fully aware of the spread of this virus. The number of patients affected by MERS in Saudi Arabia is increasing, and the Ministry of Health has reported 1291 confirmed cases and 552 deaths as of February 17, 2016 [5]. HCWs in Saudi hospitals are at risk of contracting MERS-CoV and transmitting it to family members and other contacts.

Measuring perceived risk is a challenge owing to its abstract nature. However, the concerns of HCWs and the wider community have been studied in relation to similar contagious, infectious respiratory disorders [6–8]. Investigators have explored various domains, such as self-satisfaction, in which HCWs are asked about their personal feelings of safety, anxiety, risk or threat [3, 4]. The social domain relates to the HCWs’ relationships, and includes concerns for family members and changes in social relationships [3, 4]. For HCWs, risks are associated with the workplace, so items related to support, interventions and work-related concerns are placed in the human resources domain [4, 6]. Other domains address perceptions of infection-control measures and governmental responses to diseases [7]. Distinguishing between different domains helps to identify where respondents have negative or positive perceptions, so that beneficial approaches, such as awareness campaigns and managerial support, can be established [6].

Although a number of studies have measured perceptions and knowledge in outbreaks of infection similar to that of MERS-CoV, few of these studies have focused on HCWs [9–11]. Our aims were to assess the levels of concern of HCWs in hospitals in Saudi Arabia with regard to MERS, and to identify significant predictors of high concern.

## Methods

### Study design

A cross-sectional study design was used.

### Study setting

The Saudi Ministry of National Guard Health Affairs (MNG-HA) provides health services to the military community of Saudi National Guard soldiers and their families at three King Abdulaziz Medical Cities (KAMCs) in central, eastern and western Saudi Arabia, with capacities of >1000 beds, 400 beds and 245 beds, respectively. These hospitals were of the 14 hospitals from which MERS-Cov cases were reported in Saudi Arabia, representing 3 regions of the country. All three medical cities have been registered with the Joint Commission International since 2006 and achieved accreditation in December, 2015.

Hospitals of the Ministry of National Guard in Saudi Arabia, specifically in central region showed the highest peak of infection with MERS-CoV, compared to other hospitals in the country, although there is no evidence of difference between the patient population affiliated with the National Guard and the general public. At KAMC in Riyadh, in the central region of Saudi Arabia, PHASE III of the Infectious Diseases Epidemic Plan has previously been activated, resulting in closure of the Emergency Department and Outpatient and Inpatient Services because of the MERS outbreak. Overcrowding and boarding in the Emergency Department have been identified as the main factors responsible for this situation, which has had considerable consequences on patient care. Measures have now been taken to address these issues, and the MNG-HA has established infection-control measures and practices to aggressively contain any outbreaks. The incidence of confirmed cases of MERS among the public and HCWs has varied in the three regions since the emergence of the virus in the western region in 2012. Peaks of incidence have occurred; [12, 13] in the central region in July and August 2015, when the hospital registered 58 cases of MERS-CoV infection [13]. Corresponding figures of infected HCWs in central, eastern and western medical cities were 18, 1 and 2 cases respectively.

### Subjects and sampling technique

The MNG-HA employs a large number of HCWs of various disciplines and nationalities. MNG-HA HCWs were invited to participate in this study. HCWs whose jobs entailed direct contact with patients in emergency departments and critical care units were categorized as “Direct contact group”, while those who were in contact with patient-related items, such as equipment, biological samples, as well as administrative HCWs were categorized as “Non-direct contact group”.

An appropriate sample size was calculated on the basis of results from a previous study of concerns relating to avian influenza [6], in which 82.7% of HCWs had negative perceptions of work-related risk, and the response rate was approximately 70% [6]. To compensate for dropout, incomplete questionnaires or faulty entries, a total sample size for the three regions in our study was estimated to be statistically convenient beyond 1000 study participants. A total of 1500 questionnaires were distributed among the three hospitals proportionately, based on the number of HCWs in each of these hospitals. Care was taken to ensure that participants from various fields (medicine, nursing, pharmacy, laboratory and others) were represented in the three hospitals, to minimize the possible systematic bias. All HCWs on duty during the data collection visits, in the three hospitals, were invited to participate in the study. Those who agreed to participate and who responded with completed questionnaires totaled 1031HCWs, with response rates of 62%, 65%, 68% in the three hospitals.

### Data collection

A structured, self-administered questionnaire was designed on the basis of a survey previously used in studies of the concerns of HCWs with regard to severe acute respiratory syndrome (SARS) and avian influenza pandemics [6, 8]. The survey was modified to assess HCWs’ socio-demographic and professional characteristics, attitudes and concerns regarding MERS-CoV. Questions on gender, age, marital status, level of education and professional role were included. Concerns about the disease, its severity and outcomes, and governmental efforts to combat it, were assessed using 31 concern statements. These statements were classified into five distinct domains: i) self-satisfaction (seven statements); ii) social status (six statements); iii) workplace-related (eight statements); iv) infection-control-related (five statements); and v) government-related (five statements).

Each statement had the following response choices: “strongly agree,” “agree,” “disagree,” or “strongly disagree.” A scoring system was applied using a four-point Likert scale, from no points (“strongly disagree”) to three points (“strongly agree”). The total concern scores reported by our respondents ranged from five to 80 points (from a possible range of 0–93 points). Respondents were categorized into three groups: low concern, at or below the first quartile of concern scores (in the range of 5–34 points); moderate concern, in the interquartile range of concern scores (35–45 points); and high concern, at or above the third quartile (with 46–80 points) [14].

The study investigators distributed anonymous, self-administered previously validated English-language surveys inside envelopes with cover letters to HCWs in their departments, during the period between October and December, 2014. HCWs were Saudis and expatriates of different nationalities, with Arab and non-Arab speakers, yet English language is the official language of communication among the HCWs in these hospitals. Study participants were expected to complete the survey and return it in the envelope, sealed and without identifiers. The questionnaire was pre-tested and piloted with a convenience sample of 20 HCWs who were similar in socio-demographic and professional characteristics to the members of the study population. Based on the recommendations of the pilot study, minor rewording and restyling of the questions was incorporated to simplify and improve the final questionnaire.

### Ethical issues

The survey was given to participants in sealed envelopes so that it would not be recognized by hospital staff. Participation in this study was voluntary. HCWs were assured that their feedback would not affect their performance evaluations, work status or salaries. No written consent was sought, as there were no personal identifiers on the questionnaires. The ethics committee waived the need for written consent. This study was approved by the institutional review board of the MNG-HA in Riyadh, Saudi Arabia (September 7, 2014; RC 13/243).

### Data analysis

The Statistical Package for the Social Sciences software (SPSS version 21.0; IBM Corporation, Armonk, NY, USA) was used for data analysis. Categorical socio-demographic data were summarized by frequencies and percentages of occurrence. The arithmetic mean was used as a summary statistic for concern scores, with standard deviation as a measure of dispersion. The chi-square test was used to compare frequencies of respondents at different concern levels associated with categorical variables. Means of concern scores were compared by student’s t-test (for two values) and one-way ANOVA (for more than two values). Multiple regression analyses were used to determine significant predictors of high concern scores. All two-way interactions between explanatory variables were tested and found to be non-significant. The age variable was categorized into two groups at the age of 35 years of age. This offers a simple and logical interpretation of the age variable where HCW less than the age of 35 are considered early to mid-career vs. those who are at more senior stage in their career. It also provides a roughly equal number of subjects in the two categories. For all statistical analyses, a *p* value of ≤0.05 was considered significant.

## Results

### Personal characteristics

A total of 1031 MNG-HA HCWs (304 male and 727 female) in the three regions of the Kingdom of Saudi Arabia (461, 44.7% from the central region, 361,35% from the eastern region and 209, 20.3% from the western region) agreed to participate in the study and filled questionnaires assessing their concerns regarding MERS outbreaks, with an average age of 37.1 ± 9.0 years. Approximately two-thirds (65.8%) of the participants were married, and the majority (59.1%) were nurses. Regional differences were shown in age (*p* < 0.001), nationality (*p* = 0.007), education (*p* = 0.003) and job titles (*p* < 0.001) of HCWs, as well as their contact with patients (*p* = 0.005) (Table 1).

**Table 1:** Sociodemographic characteristics of HCWs at Ministry of National Guard-Health Affairs in different regions of Saudi Arabia

	Eastern region no.(%)	Central region no.(%)	Western region no.(%)	Total no.(%)
	361 (35.0)	461 (44.7)	209 (20.3)	1031 (100.0)
Gender				
Male	110 (30.5)	127 (27.5)	67 (32.1)	304 (29.5)
Female	251 (69.5)	334 (72.5)	142 (67.9)	727 (70.5)
	χ^2^ = 1.665, df = 2, *p* = 0.435			
Age (years)				
≤ 35	149 (41.3)	239 (51.8)	72 (34.4)	460 (44.6)
> 35	212 (58.7)	222 (48.2)	137 (65.6)	571 (55.4)
x ± SD	38.0 ± 9.4	35.4 ± 8.3	39.3 ± 9.2	37.1 ± 9.0
	χ^2^ = 20.119, df = 2, *p* < 0.001*			
Marital Status				
Single	110 (30.5)	172 (37.3)	71 (34.0)	353 (34.2)
Married	251 (69.5)	289 (62.7)	138 (66.0)	678 (65.8)
	χ^2^ = 4.214, df = 2, *p* = 0.122			
Nationality				
Saudi	72 (19.9)	136 (29.5)	50 (23.9)	258 (25.0)
Non Saudi	289 (80.1)	325 (70.5)	159 (76.1)	773 (75.0)
	χ^2^ = 10.024, df = 2, *p* = 0.007*			
Education Level				
BS	334 (72.5)	234 (64.8)	135 (64.6)	703 (68.2)
Diploma	87 (18.9)	83 (23.0)	36 (17.2)	206 (20.0)
MS/PHD	40 (8.6)	44 (12.2)	38 (18.2)	122 (11.8)
	χ^2^ = 15.98, df = 4, *p* = 0.003*			
Job title				
Physician	93 (25.8)	53 (11.5)	34 (16.3)	180 (17.5)
Nursing	202 (56.0)	294 (63.8)	113 (54.1)	69 (59.1)
Technician	60 (16.6)	47 (10.2)	45 (21.5)	152 (14.7)
Pharmacy	5 (1.3)	35 (7.6)	12 (5.7)	52 (5.0)
Administrative	1 (0.3)	32 (6.9)	5 (2.4)	38 (3.7)
	χ^2^ = 82.185, df = 8, *p* < 0.001*			
Direct patient contact				
Yes	331 (91.7)	411 (89.2)	173 (82.8)	915 (88.7)
No	30 (8.3)	50 (10.8)	36 (17.2)	116 (11.3)
	χ^2^ = 10.672, df = 2, *p* = 0.005*			

*χ2* Pearson Chi-square test, *df* degree of freedom, *BS* Bachelor of Science, *MS* Master of Science, *PHD* Doctor of Philosophy

### Concerns of HCWs regarding MERS outbreaks

The responses to the 31 items in the questionnaire varied considerably, from a high of 87.9% agreeing that they did not feel safe at work using standard precautions to a low of 7.1% agreeing that they felt they should change job because of the MERS crisis (Table 2). Although 70.4% of HCWs responded that they felt at risk of contracting the MERS-CoV infection at work, only 35.5% agreed that this risk was absolute, and only 36.8% agreed that they felt unsafe in the workplace. More than half of respondents (60.9%) agreed that they felt obliged to care for affected patients and 69.1% agreed that they would feel threatened if a colleague became infected. Less than half of respondents agreed with other items in the self-satisfaction, social, workplace and infection-control domains.

**Table 2:** Responses of health-care workers to concern statements about the MERS outbreak in Saudi Arabia

	Agree/Strongly agree n (%)	Disagree/Strongly disagree n (%)
A. Self-satisfaction domain		
1. I feel unsafe working at my workplace.	379 (36.8)	652 (63.2)
2. I feel anxious while working with a febrile patient.	434 (42.1)	597 (57.9)
3. I feel at risk to contract a MERS-CoV infection at work.	726 (70.4)	305 (29.6)
4. I feel obliged to care for a MERS-CoV-infected patient.	628 (60.9)	403 (39.1)
5. I feel hopeless I might eventually get a MERS-CoV at work.	366 (35.5)	665 (64.5)
6. I feel threatened if one of my colleagues contracted MERS-CoV.	712 (69.1)	319 (30.9)
7. If I get MERS-CoV, I don’t feel confident an employee will care for me?	345 (33.5)	686 (66.5)
B. Social status-related domain		
1. I feel that I should limit my social activities due to MERS-CoV.	402 (39.0)	629 (61.0)
2. I feel I will transmit MERS-CoV to my family members.	458 (45.1)	573 (54.9)
3. I feel that my family members avoid me since I work in hospital.	106 (10.3)	925 (89.7)
4. I feel I should avoid leaving my home due to MERS-CoV.	167 (16.2)	864 (83.8)
5. I feel my family will not look after me if I was infected.	89 (8.6)	942 (91.4)
6. I don’t feel confident telling my family and friends if I was infected.	181 (17.6)	850 (82.4)
C. Workplace-related domain		
1. I feel that my institution didn’t support me during the MERS-CoV crisis.	163 (15.8)	868 (84.2)
2. I feel that my institution is losing control of the MERS-CoV crisis.	135 (13.1)	896 (86.9)
3. I feel overwhelmed with the new MERS-CoV regulations.	491 (47.6)	540 (52.4)
4. I feel MERS-CoV crisis increased my workload.	377 (36.6)	654 (63.4)
5. I feel that the increase in workload was not meet with proper staffing.	502 (48.7)	529 (51.3)
6. I feel absence from work reduces the chance of getting MERS-CoV.	169 (16.4)	862 (83.6)
7. In case I had MERS-CoV, I feel ashamed telling my manager/colleagues.	93 (9.0)	938 (91.0)
8. I feel I should change my current job due to MERS-CoV crisis.	73 (7.1)	958 (92.9)
D. Infection control-related domain		
1. I am not confident with the current infection control measures.	151 (14.6)	880 (85.4)
2. I don’t feel proper infection control training has been offered to me.	219 (21.2)	812 (78.8)
3. I don’t feel an infection specialist is accessible to respond to my concerns.	141 (13.7)	890 (86.3)
4. I don’t feel there is MERS-CoV outbreak plan set at my area.	296 (28.7)	735 (71.3)
5. I don’t feel safe at work when I use the standard precautions.	906 (87.9)	125 (12.1)
E. Government-related domain		
1. I feel the government should restrict travel from /to areas of disease.	760 (73.7)	271 (26.3)
2. I feel the government should isolate MERS-CoV cases in special hospitals	904 (87.7)	127 (12.3)
3. I feel government should avoid inviting expatriates infected areas.	673 (65.3)	358 (34.7)
4. I feel schools and shopping markets need to be closed to control MERS-CoV.	196 (19.0)	835 (81.0)
5. I don’t feel MERS-CoV has been highlighted and discussed efficiently in media.	245 (23.8)	786 (76.2)

*Abbreviation*: *MERS-CoV* Middle East Respiratory Syndrome coronavirus

The majority of HCWs questioned (87.7%) agreed that the government should isolate patients with MERS in specialized hospitals, 73.7% agreed that it should restrict travel to and from areas with the disease and 65.3% agreed that it should avoid inviting expatriates from such areas. However, those who agreed that schools and markets need to be closed constituted only 19% of participants (Table 2).

Overall, 25.1% of HCWs were classified as having high concern, 48.0% moderate concern and 26.9% low concern. The mean concern score was 40.2 ± 11.0, out of a maximum possible concern score of 93. Chi-square analyses (Table 3) showed that the distribution of HCWs into categories of low, moderate or high concern was not affected by gender (χ^2^ = 1.69; *p* = 0.43) or marital status (χ^2^ = 1.998; *p* = 0.368), but it was affected by age (χ^2^ = 19.747; *p* <0.001), nationality (χ^2^ = 19.409; *p* <0.001), education (χ^2^ = 15.654; *p* = 0.004), occupation (χ^2^ = 20.697; *p* = 0.008), geographical location (χ^2^ = 37.290; *p* <0.001) and direct patient contact (χ2 = 9.122, *p* = 0.010).

**Table 3:** Levels of concern regarding the MERS outbreaks in health-care workers in Saudi Arabia according to personal characteristics

Characteristics	High concern (score = 46–80)	Moderate concern (score = 35–45)	Low concern (score = 5–34)	Mean concern score
Total	259 (25.1)	495 (48.0)	277 (26.9)	40.2 ± 11.0
Gender				
Male	83 (27.3)	137 (45.1)	84 (27.6)	39.9 ± 11.8
Female	176 (24.2)	358 (49.2)	193 (26.6)	40.3 ± 10.6
	χ^2^ = 1.690, *p* = 0.430			*t* = −0.480, *p* = 0.631
Age				
≤35	106 (23.0)	199 (43.3)	155 (33.7)	41.4 ± 11.3
>35	153 (26.8)	296 (51.8)	122 (21.4)	39.3 ± 10.6
	χ2 = 19.747, *p* < 0.001*			*t* = 3.035, *p* = 0.002*
Marital status				
Unmarried	88 (24.9)	161 (45.6)	104 (29.5)	40.3 ± 10.7
Married	171 (25.2)	334 (49.3)	173 (25.5)	40.2 ± 11.1
	χ^2^ = 1.998, *p* = 0.368			*t* = −0.096, *p* = 0.923
Nationality				
Saudi	51 (19.8)	111 (43.0)	96 (37.2)	43.0 ± 11.7
Non-Saudi	208 (26.9)	384 (49.7)	181 (23.4)	39.3 ± 10.5
	χ^2^ = 19.409, *p* < 0.001*			*t* = 4.567, *p* < 0.001*
Level of education				
Diploma	44 (21.4)	103 (50.0)	59 (28.6)	40.7 ± 11.2
BS	168 (23.9)	338 (48.1)	197 (28.0)	40.5 ± 10.6
MSN/PHD	47 (38.5)	54 (44.3)	21 (17.2)	37.5 ± 12.1
	χ^2^ = 15.654, df = 2, *p* = 0.004*			F = 4.148, df = 2, *p* = 0.016*
Job title				
Physician	63 (35.0)	83 (46.1)	34 (18.9)	37.3 ± 10.9
Nurse	149 (24.5)	286 (47.0)	174 (28.5)	40.6 ± 10.9
Technician	31 (20.4)	84 (55.3)	37 (24.3)	40.6 ± 10.5
Pharmacist	10 (19.2)	25 (48.1)	17 (32.7)	42.7 ± 10.7
Administrative	6 (15.8)	17 (44.7)	15 (39.5)	43.9 ± 10.8
	χ^2^ = 20.697, df = 4, *p* = 0.008*			F = 5.266, df = 4, *p* < 0.001*
Geographical region of employment				
Eastern	125 (34.6)	152 (42.1)	84 (23.3)	37.9 ± 11.5
Central	88 (19.1)	223 (48.4)	150 (32.5)	42.2 ± 10.8
Western	46 (22.0)	120 (57.4)	43 (20.6)	39.9 ± 9.4
	χ^2^ = 37.290, df = 2, *p* < 0.001*			F = 15.822, df = 2, *p* < 0.001*
Direct patient contact				
Yes	237 (25.9)	424 (46.3)	254 (27.8)	40.2 ± 11.1
No	22 (19.0)	71 (61.2)	23 (19.8)	40.4 ± 9.6
	χ^2^ = 9.122, *p* = 0.010*			*t* = −0.188, *p* = 0.851

*χ2* Pearson Chi squared test, *LT* Chi square test for linear trend, *f* Analysis of variance (ANOVA) test, *df* degree of freedom

*Statistically significant difference

After adjustment for covariates in multiple regression analyses (Table 4), high overall concern scores were independently associated with working in the central region (*p* < 0.001), Saudi nationality (*p* < 0.001) and not being a physician (*p* < 0.001). Working in central region was a significant predictor of high concern score in self-satisfaction (*p* < 0.001), work place (*p* < 0.001) and government-related domains (*p* = 0.021). Not being a physician was a significant predictor of high concern score in self-satisfaction (*p* = 0.001), social status (*p* = 0.003), workplace (*p* = 0.013) and government-related (*p* = 0.009) domains. Saudi nationality was a significant predictor of high concern score in self-satisfaction, workplace and infection control-related domains (*p* < 0.001 each). High concern scores of government-related domain was predicted by younger age HCWs (*p* = 0.008). HCWs of lower education showed significantly lower concern scores of infection control- but higher score for social status-related domains (*p* = 0.043 & *p* = 0.045 respectively).

**Table 4:** Predictors of high concern scores among health-care workers in Saudi Arabia

	Overall concern	Self-satisfaction domain	Social status related domain	Work place related domain	Infection control related domain	Government related domain
	β (t-value.) *P*-value	β (t-value.) *P*-value	β (t-value.) *P*-value	β (t-value.) *P*-value	β (t-value.) *P*-value	β (t-value.) *P*-value
Gender	0.56 (0.67)	0.01 (0.01)	0.14 (0.57)	0.37 (1.31)	0.26 (1.39)	−0.21 (−1.28)
Male vs. Female [ref.]	*p* = 0.500	*P* = 0.995	*P* = 0.572	*P* = 0.189	*P* = 0.165	*P* = 0.199
Age in years	−0.81 (−1.07)	−0.21 (−0.77)	0.07 (0.30)	0.02 (0.09)	−0.30 (−1.77)	−0.39 (−2.65)
> 35 vs ≤35 [ref.]	*p* = 0.286	*P* = 0.445	*P* = 0.763	*P* = 0.926	*P* = 0.077	*P* = 0.008*
Marital status	0.88 (1.16)	0.09 (0.31)	0.35 (1.57)	−0.01 (−0.01)	0.21 (1.22)	0.24 (1.64)
Married vs. Single [ref.]	*P* = 0.245	*P* = 0.758	*P* = 0.118	*P* = 0.995	*P* = 0.224	*P* = 0.102
Nationality	3.92 (4.65)	1.33 (4.33)	0.06 (0.25)	1.13 (3.99)	1.22 (6.43)	0.17 (1.06)
Saudi vs Non Saudi [ref.]	*P* < 0.001*	*P* < 0.001*	*P* = 0.801	*P* < 0.001*	*P* < 0.001*	*P* = 0.291
Education	1.37 (1.16)	0.29 (0.68)	0.71 (2.01)	0.53 (1.34)	−0.54 (−2.03)	0.38 (1.66)
Lower vs. higher [ref.]	*P* = 0.248	*P* = 0.494	*P* = 0.045*	*P* = 0.180	*P* = 0.043*	*P* = 0.098
Job	−3.89 (−3.68)	−1.33 (−3.44)	−0.93 (−2.98)	−0.88 (−2.50)	−0.20 (−0.92)	−0.53 (−2.61)
Physicians vs. others [ref.]	*P* < 0.001*	*P* = 0.001*	*P* = 0.003*	*P* = 0.013*	*P* = 0.359	*P* = 0.009*
Direct contact	0.01 (0.021)	0.46 (1.18)	−0.29 (−0.94)	−0.20 (−0.59)	−0.04 (−0.18)	0.09 (0.47)
Yes vs. None [ref.]	*P* = 0.993	*P* = 0.238	*P* = 0.346	*P* = 0.562	*P* = 0.856	*P* = 0.636
Region	2.67 (3.91)	1.09 (4.39)	0.14 (0.69)	0.91 (3.99)	0.23 (1.47)	0.30 (0.47)
Central vs. others [ref.]	*P* < 0.001*	*P* < 0.001*	*P* = 0.492	*P* < 0.001*	*P* = 0.142	*P* = 0.021*
Constant	37.202 (22.05) *P* < 0.001*	9.58 (15.55) *P* < 0.001*	5.59 (11.17) *P* < 0.001*	7.83 (13.89) *P* < 0.001*	6.49 (17.1) *P* < 0.001*	7.72 (23.75) *P* < 0.001*

*β* beta coefficient, *t* t statistic

*significant association

## Discussion

Health-care institutions are expected to have a major role during a pandemic [15], when HCWs are at a high risk of exposure and infection [16]. During the most recent outbreak of SARS, HCWs suffered considerable stress, partly from an overstretched health-care system [17, 18]. A similar scenario is expected should a MERS outbreak occur in Saudi Arabia. Our results show that the attitude of a sample of HCWs in Saudi Arabia toward MERS-CoV infection is in the negative range, with an overall average concern score of 40 out of a maximum possible score of 93 points, indicating a moderate level of concern. In a study conducted in a tertiary teaching hospital in Greece, more than half of the surveyed HCWs experienced moderately high levels of worry about the A/H1N1 influenza pandemic [19]. In our study, one-fourth of HCWs had high levels of concern regarding the MERS outbreak. This finding is in accordance with the results of a similar study, published in 2015, of HCWs at Makkah hospitals, Saudi Arabia [10]. High levels of concern have also been demonstrated in the Saudi public [20]. These negative attitudes and high levels of concern could be attributed to the novelty of MERS-CoV infection and the lack of previous experience with, or exposure to, MERS. However, perceptions are not always negative, and results published in 2014 from a study in the Al Qassim region of Saudi Arabia demonstrated positive attitudes in HCWs towards MERS [11]. There has been possibility that media coverage may be influencing HCWs’ attitudes to MERS-CoV.

When comparing our findings with those from other studies, other factors that might contribute to any observed differences should be taken into consideration. Prominent among these are the variability in definition of high concern (cut-off point beyond which the high level of concern is considered), age difference between studied subjects and time of the study. Lack of standardization in methodology between different studies creates difficulties for making proper comparisons between different populations.

An important finding in our study was that high level of concern was prevalent among HCWs, although it took different forms. Concern was mainly observed when respondents replied negatively to questions regarding fears of infection of a family member, risk of infection if one of the colleagues gets infected, risks associated with dealing with a febrile patient, or obligation of care provision for patients infected with MERS-CoV and lack of faith in standard precautions. Similar responses have been recorded in relation to MERS and other diseases. More than half (55%) of surveyed HCWs in Japan indicated a high level of fear and anxiety of SARS-CoV infection, even in the absence of an epidemic, and a high proportion (92%) preferred to avoid patients with SARS [21]. Approximately 90% of surveyed HCWs in Thailand accepted the personal risk of caring for patients with H5N1 infections [22], and approximately 78% of the Saudi public who were surveyed agreed that schools should close in case of an H1N1 influenza epidemic [7]. However, this finding was not in agreement with the finding of the present study where only 19% of HCWs agreed that schools and shopping markets need to be closed.

MERS-CoV is continuing to spread to countries outside the Middle East, and MERS remains a public-health risk. The possible consequences of this spread are serious in view of the pattern of nosocomial transmission. Five days after the publication of a WHO report in May 2015 [23] denying the possibility of sustained outward transmission to persons in close contact with those affected by MERS, on aircraft or in countries outside the Middle East, the first case of a MERS-CoV infection was reported in Seoul, South Korea. [24] In our study, the majority of participants agreed that the government should isolate patients with MERS in special hospitals, that it should avoid inviting expatriate workers from areas where the disease is prevalent, that it should restrict travel to and from such areas, and that they felt at risk of contracting a MERS-CoV infection at work. Further spread of the virus to countries with poorly developed health-care systems and laboratory facilities, in which an unexpected virus cannot be rapidly identified, may result in a widespread outbreak or epidemic; this description applies to many of the 182 countries from which Ramadan, Hajj and Umrah pilgrims originate.

The WHO has highlighted the importance of preparedness plans in reducing the effect of outbreaks [25]. The MNG-HA has developed a plan documenting the medical and public-health responses to a MERS outbreak. The plan describes health-care institutions as vital components in an outbreak, and makes provisions to protect HCWs through infection-control measures and personal-protection practices. However, our results show that the majority of HCWs feel unsafe at work when using the standard infection-control precautions. Moreover, the majority feel at risk of contracting a MERS-CoV infection at work. This result is similar to the findings of a study of doctors in the UK, in which approximately two-thirds felt that their health-care system would have problems coping with a pandemic [26]. Ensuring that adequate protective measures are in place could provide a measure of reassurance to HCWs. The provision of knowledge and skills could help HCWs to feel better prepared and maintain staff morale during an outbreak.

The complex situation of the MERS outbreak in Saudi Arabia highlighted the importance of some cultural issues; such as the strong ties with family members, relatives and friends, which means that Saudis are likely to visit and care for loved ones who are afflicted with MERS [27]. This cultural issue may partly explain the higher concern among Saudi HCWs compared with non-Saudis. Non-Saudi HCWs, as expatriate workers, are more likely to be single and/or to have family members living outside Saudi Arabia, so they might have less immediate concern about transmitting the disease. Saudi nationals are more likely to be exposed to local media, or more likely to be critical of their own government’s policies than foreign nationals.

Our results show that physicians have less concern than other HCWs in relation to MERS. This finding was evident in all domains except for infection control-related domain. This finding differed from the results of previous studies [10, 11, 22, 28]. A low level of concern among physicians could be attributed to their greater opportunities for professional development and clinical training compared with other HCWs, along with possible previous experience of similar diseases with infectious viral origins, such as SARS and swine flu. Clinicians may have more access to professional journals, whereas others may obtain more information from mass media. Knowledge and experience could result in positive attitudes that can be explained by the theory of reasoned action, which predicts that behavioral intent is caused by both attitudes and subjective norms. [29]

In emergency situations, HCWs face conditions that lead to physical and mental exhaustion [6]. The critical situation in central region, where PHASE III of the Infectious Diseases Epidemic Plan has previously been activated, resulting in closure of the Emergency Department and Outpatient and Inpatient Services because of the MERS outbreak, is likely to have affected the perception and attitudes of HCWs in the central region more than in other regions of the Kingdom. Our results show that location in the central region is associated with higher concern scores in relation to MERS compared with location elsewhere, even after adjusting for covariates. This finding was evident for self-satisfaction, workplace and government-related domains. The experience of the effects of the outbreak in the central region could have contributed to this elevated level of concern.

### Limitations

Because this study had a cross-sectional design, relationships between the predictor variables and the dependent variable (the concern score) can only be described as general associations rather than causal relationships. As with any survey based on a self-administered questionnaire, the self-reported information on which the analysis and interpretations are based may not be entirely accurate, mainly because of the possibility of recall bias of HCWs giving a more positive response than would be revealed by other data-collection methods. The study might be subjected to a selection bias due the possibility of not having all disciplines involved equally. However, the response rate ranged from 62 to 68% in the three regions, a finding that should not reflect a high level of systematic bias, if any. Moreover, the respondents were all HCWs in tertiary military hospitals, and the results might not reflect the concerns of all HCWs, or those in non-military hospitals in Saudi Arabia. In addition, the design process for the questionnaire did not include a qualitative focus-group discussion to investigate the attitudes of HCWs in depth. Despite the identified limitations, these results contribute to the information relating to a major health problem faced by HCWs in Saudi Arabia, especially in the MNG-HA. Very little research has previously been carried out in this field.

## Conclusions

These results can provide a reference point for the monitoring of the perceptions of HCWs in the event of a future outbreak of infectious disease in Saudi Arabia. The majority of respondents in this study were concerned about their risk of exposure to illness at work. This level of concern could have an adverse effect on the management of suspected or confirmed cases of MERS, and on the overall effectiveness of HCWs during the outbreak. The level of concern was high in Saudi nationals and non-physicians, and in HCWs in the central region of Saudi Arabia, who have previously experienced disease-control measures.

Compliance with the recommendations of the WHO [23] is necessary to ensure adequate support for front-line HCWs. Provisions should be made to protect them through infection-control measures, personal-protection practices and anti-viral medications.

Measures to enhance protection for HCWs and to minimize the psychological effect of the perceived risk of infection should be addressed in the planning stage prior to any future outbreak. These measures could take the form of counseling and incentives to boost morale and maintain levels of service, as well as education. All of these measures could be crucial to maintaining the integrity of the health-care system during an outbreak.

## Abbreviations

HCWs: Health care workers
IRB: Institutional Review Board
KAMC: King Abdulaziz Medical city
MERS-CoV: Middle East Respiratory Syndrome-corona virus
MNG-HA: Ministry of National Guard-Health Affairs
PMS: Percentage mean score
WHO: World Health Organization

## References

[1] de Groot RJ, Baker SC, Baric RS, Brown CS, Drosten C, Enjuanes L, Fouchier RA, Galiano M, Gorbalenya AE, Memish ZA, Perlman S (2013). Middle East respiratory syndrome coronavirus (MERS-CoV): announcement of the Coronavirus Study Group. J Virol.

[2] Zaki AM, Van Boheemen S, Bestebroer TM, Osterhaus AD, Fouchier RA (2012). Isolation of a novel coronavirus from a man with pneumonia in Saudi Arabia. N. Engl. J. Med..

[3] WHO MERS-CoV: Global Summary and risk assessment, 5 December 2016 WHO/MERS/RA/16.1. http://www.who.int/emergencies/mers-cov/mers-summary-2016.pdf?ua=1. Accessed 11 Dec 2016.

[4] State of Knowledge and Data Gaps of Middle East Respiratory Syndrome Coronavirus (MERS-CoV) in Humans. Who Mers-Cov Research Group. PLoS Curr. 2013. doi:10.1371/currents.outbreaks.0bf719e352e7478f8ad85fa30127ddb8. 10.1371/currents.outbreaks.0bf719e352e7478f8ad85fa30127ddb8 PMC3828229 24270606

[5] Ministry of Health, Command & Control Center, Saudi Arabia. http://www.moh.gov.sa/en/ccc/Pages/default.aspx. Accessed 10 Dec 2016.

[6] Wong TY, Koh GC, Cheong SK, Lee HY, Fong YT, Sundram M, Koh K, Chia SE, Koh D (2008). Concerns, perceived impact and preparedness in an avian influenza pandemic--a comparative study between healthcare workers in primary and tertiary care. Ann-Acad Med Singapore.

[7] Balkhy HH, Abolfotouh MA, Al-Hathlool RH, Al-Jumah MA (2010). Awareness, attitudes, and practices related to the swine influenza pandemic among the Saudi public. BMC Infect Dis.

[8] Koh D, Lim MK, Chia SE, Ko SM, Qian F, Ng V, Tan BH, Wong KS, Chew WM, Tang HK, Ng W (2005). Risk perception and impact of Severe Acute Respiratory Syndrome (SARS) on work and personal lives of healthcare workers in Singapore: what can we learn?. Med Care.

[9] Albano L, Matuozzo A, Marinelli P, Di Giuseppe G (2014). Knowledge, attitudes and behaviour of hospital health-care workers regarding influenza A/H1N1: a cross sectional survey. BMC Infect Dis.

[10] Nour MO, Babilghith AO, Natto HA, Al-Amin FO, Alawneh SM (2015). Knowledge, attitude and practices of healthcare providers towards MERS-CoV infection at Makkah hospitals, KSA. Int Res J Med Med Sci.

[11] Khan MU, Shah S, Ahmad A, Fatokun O (2014). Knowledge and attitude of healthcare workers about middle east respiratory syndrome in multispecialty hospitals of Qassim, Saudi Arabia. BMC Public Health.

[12] Alameer K, Abukhzam B, Khan W, El-Saed A, Balkhy H (2015). Middle East respiratory syndrome coronavirus (MERS-Cov) screening of exposed healthcare workers in a tertiary care hospital in Saudi Arabia. Antimicrob Resist Infect Control.

[13] Balkhy HH, Alenazi TH, Alshamrani MM (2016). Notes from the field: nosocomial outbreak of middle east respiratory syndrome in a large tertiary care hospital — Riyadh, Saudi Arabia, 2015. MMWR Morb Mortal Wkly Rep.

[14] Abolfotouh MA, Al-Alwan IA, Al-Rowaily MA. Prevalence of metabolic abnormalities and association with obesity among Saudi college students. Int J Hypert. 2012. Article ID 819726, 8 pages. Doi:10.1155/2012/819726. 10.1155/2012/819726 PMC3536048 23316346

[15] Ministry of Health, Singapore. Influenza Pandemic Readiness and Response Plan. Available at: https://www.moh.gov.sg/content/dam/moh_web/PressRoom/Current_Issues/2009/Main%20Document%20Public%20_Jan09.pdf. Accessed 11 Dec 2016.

[16] Wilson N, Baker M, Crampton P, Mansoor O (2005). The potential impact of the next influenza pandemic on a national primary care medical workforce. Hum. Resour. Health.

[17] Tam CW, Pang EP, Lam LC, Chiu HF (2004). Severe acute respiratory syndrome (SARS) in Hong Kong in 2003: stress and psychological impact among frontline healthcare workers. Psychol Med.

[18] Koh D, Lim MK, Chia SE (2003). SARS: health care work can be hazardous to health. Occup Med.

[19] Goulia P, Mantas C, Dimitroula D, Mantis D, Hyphantis T (2010). General hospital staff worries, perceived sufficiency of information and associated psychological distress during the A/H1N1 influenza pandemic. BMC Infect Dis.

[20] Almutairi KM, Al Helih EM, Moussa M, Boshaiqah AE, Alajilan AS, Vinluan JM, Almutairi A (2015). Awareness, attitudes, and practices related to coronavirus pandemic among public in Saudi Arabia. Fam Community Health.

[21] Imai T, Takahashi K, Hasegawa N, Lim MK, Koh D (2005). SARS risk perceptions in healthcare workers. Japan Emerg Infect Dis.

[22] Apisarnthanarak A, Phattanakeitchai P, Warren DK, Fraser VJ (2008). Impact of knowledge and positive attitudes about avian influenza (H5N1 virus infection) on infection control and influenza vaccination practices of Thai healthcare workers. Infect. Control Hosp. Epidemiol..

[23] Ben EP, Van Kerkhove MD (2015). Middle East respiratory syndrome coronavirus (MERS-CoV): current situation 3 years after the virus was first identified. Relevé épidémiologique hebdomadaire/Section d’hygiène du Secrétariat de la Société des Nations = Weekly epidemiological record/Health Section of the Secretariat of the League of Nations.

[24] Middle East respiratory syndrome coronavirus (MERS-CoV) – Republic of Korea. Disease outbreak news. 25 October 2015. http://www.who.int/csr/don/25-october-2015-mers-korea/en/. Accessed 10 Dec 2016.

[25] World Health Organization. The World Health Report 2006 – Working Together for Health. Available at: http://www.who.int/whr/2006/en/. Accessed 11 Dec 2016.

[26] Cole A (2006). Two thirds of doctors in UK say the NHS could not cope with bird flu epidemic. BMJ: Brit Med J.

[27] Almutairi AF, Adlan AA. A Closer Look at the Middle Eastern Respiratory Syndrome (MERS-CoV) Outbreak in Saudi Arabia. J Nurs Care. 2015;4(305):2167–1168.

[28] Gizaw GD, Alemu ZA, Kibret KT (2015). Assessment of knowledge and practice of health workers towards tuberculosis infection control and associated factors in public health facilities of Addis Ababa, Ethiopia: A cross-sectional study. Arch Public Health.

[29] Fisher WA, Fisher JD, Rye BJ (1995). Understanding and promoting AIDS-preventive behavior: insights from the theory of reasoned action. Health Psychol.

